# Absolute Ranging with Time Delay Interferometry for Space-Borne Gravitational Wave Detection

**DOI:** 10.3390/s24072069

**Published:** 2024-03-24

**Authors:** Dan Luo, Mingyang Xu, Panpan Wang, Hanzhong Wu, Chenggang Shao

**Affiliations:** 1 MOE Key Laboratory of Fundamental Physical Quantities Measurement, Hubei Key Laboratory of Gravitation and Quantum Physics, PGMF, School of Physics, Huazhong University of Science and Technology, Wuhan 430074, China; m202170204@hust.edu.cn (D.L.); mingyangxu@hust.edu.cn (M.X.); ppwang@hust.edu.cn (P.W.); 2 State Key Laboratory of Applied Optics, Changchun Institute of Optics, Fine Mechanics and Physics, Chinese Academy of Sciences, Changchun 130033, China

**Keywords:** gravitational wave detection, laser phase noise, arm locking, Michelson interferometer

## Abstract

In future space-borne gravitational wave (GW) detectors, time delay interferometry (TDI) will be utilized to reduce the overwhelming noise, including the laser frequency noise and the clock noise etc., by time shifting and recombining the data streams in post-processing. The successful operation of TDI relies on absolute inter-satellite ranging with meter-level precision. In this work, we numerically and experimentally demonstrate a strategy for inter-satellite distance measurement. The distances can be coarsely determined using the technique of arm-locking ranging with a large non-ambiguity range, and subsequently TDI can be used for precise distance measurement (TDI ranging) by finding the minimum value of the power of the residual noises. The measurement principle is introduced. We carry out the numerical simulations, and the results show millimeter-level precision. Further, we perform the experimental verifications based on the fiber link, and the distances can be measured with better than 0.05 m uncertainty, which can well satisfy the requirement of time delay interferometry.

## 1. Introduction

In 2015, the GWs predicted by Einstein [1,2,3,4,5,6,7] were first observed by the ground-based GW detector, the laser interferometer gravitational-wave observatory LIGO [8,9,10] and Virgo [11]; this was of key importance for physics and cosmology. Nonetheless, limited by ground vibration and the gravity gradient, ground-based GW detectors can only measure the GWs in the band from 10 Hz to tens of kHz [12]. For the sake of the observation of low-frequency GWs from 0.1 mHz to 1 Hz, space-borne GW detectors have been proposed, which are composed of three spacecraft flying in an equilateral-triangle constellation. LISA [13,14] will be the first space-borne GW detector, and other LISA-like detectors include TAIJI [15] and TianQin [16]. The GW signals, which are the oscillation of space and time, can be detected via heterodyne interferometry by exchanging the laser beams between the spacecraft. However, GW signals are often very weak at about 10^−20^ [1] in the science band, and therefore noise management is required to reduce the overwhelming noises made primarily by laser frequency and the clock [17,18]. Time delay interferometry [19,20,21] (TDI) was therefore proposed in the post-process to reduce the laser frequency noise and the clock noise, aiming to highlight the GW signals.

TDI is a kind of post-processing technique, in which the data streams are time-shifted and recombined to form a virtual equal-arm interferometer [22,23]. Consequently, the laser frequency noise and the clock noise can be canceled out by beating the corresponding data streams based on various TDI combinations. In this case, the inter-spacecraft distances should be pre-determined for the implementation of TDI. Please note that the ultimate performance of TDI is determined by the precision of the inter-spacecraft distance measurement. The technique of pseudorandom noise ranging (PRNR) [24,25,26] can be used to precisely determine the inter-spacecraft distances, where a pseudorandom sequence is encoded onto the laser beams based on an electro-optic phase modulator with low modulation depth. The distances can be measured by the peak position of the autocorrelation with meter-level precision. However, there can be a slight difference between the result of PRNR and the real inter-satellite distance [27]. Meanwhile, an electro-optic modulator is needed to phase-modulate the optical carrier, leading to additional hardware requirements and power consumption. On the other hand, the non-ambiguity range of the distance measurement is limited by the code length of the pseudorandom sequence, which is often hundreds of km; much less than the baseline length of the space-borne GW detectors. In general, the deep space network can be used to coarsely determine the inter-satellite distances with a large non-ambiguity range. However, the deep space network is not always available because of its busy schedule. TDI ranging is an alternative method to measure inter-spacecraft distances, which potentially relies on the flight time of the laser frequency noises. In 2005, TDI ranging [27] was first proposed and tested in the software of Synthetic LISA. The results show that TDI ranging can support the operation of TDI, and retain the GW signals. In the case of the experimental demonstration, the hardware-based LISA simulator utilizes the electrical delay units to introduce the multi-second phase delays, which can further verify the performance of TDI ranging and TDI [28]. Nevertheless, the demonstration of the electrical delay method is ideal, resulting in the possible loss of some optical characteristics.

In this work, we numerically and experimentally demonstrate that TDI ranging can realize inter-satellite distance measurement with high precision. In particular, we propose a novel scheme for inter-satellite ranging. We use the technique of arm-locking [29,30,31] ranging to pre-determine the inter-satellite distance and consequently the non-ambiguity range can be expanded to be extremely large. We perform numerical simulations and experimental demonstrations to examine the performance of TDI ranging. In the experiments, we develop a fiber-based setup to measure the optical delays. The results show that the capability of TDI ranging can well meet the requirement of TDI.

## 2. Concept of Time Delay Interferometry

**Figure 1:**
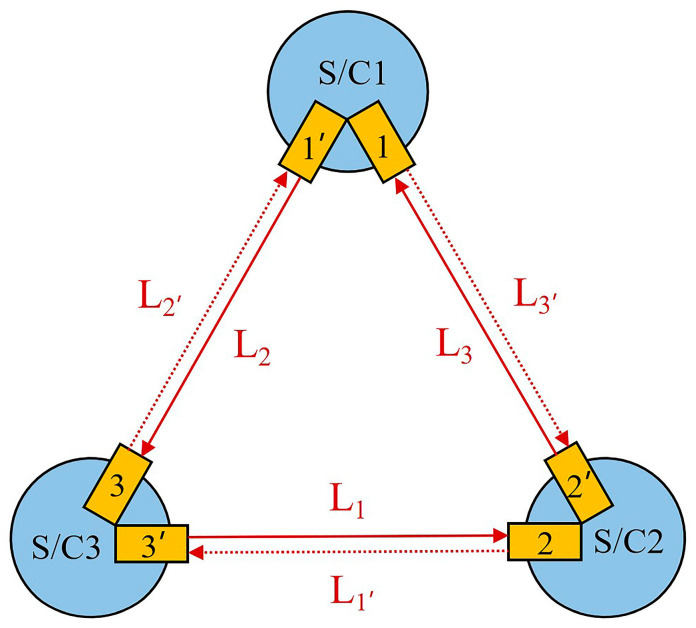
Triangle configuration for space-borne gravitational wave detector. S/C: spacecraft; *L_i_* means the light travels counterclockwise, and *L_i_*_′_ means the light propagates clockwise. There are three spacecraft in the space-borne GW detector, and each spacecraft hosts two lasers working as the signal source. The six lasers are mutually locked in a specific frequency plan, and the whole constellation shares one cavity-stabilized laser equivalently.

LISA will be the first space-borne GW detector consisting of a nearly equilateral triangle constellation with about 2.5 × 10^6^ km baseline (i.e., 8.3 s light travel time), as shown in Figure 1. *L_i_* means the light travels counterclockwise, and *L_i_*_′_ means the light propagates clockwise; *i* = 1, 2, 3. The GWs can be measured by the laser heterodyne interferometry between the satellites, where the drag-free proof masses serve as the endpoints of the large-scale interferometers. Each spacecraft hosts two laser sources at about 1064 nm on two optical benches, and one is the master laser. The laser power will be boosted to several watts to travel the long baseline. Considering the inter-satellite situation, the optical transponder scheme is recommended to maintain the signal to noise ratio and the distant laser will be well-locked to the incoming beam despite the low optical power. Therefore, one laser works as the master laser while all the other lasers are offset phase locked to this master laser in the specific plans. In this case, the whole constellation shares one cavity-stabilized laser equivalently. It is worthwhile noting that different locking schemes (also referred to as frequency plans) can be used, with consideration of all six lasers.

**Figure 2:**
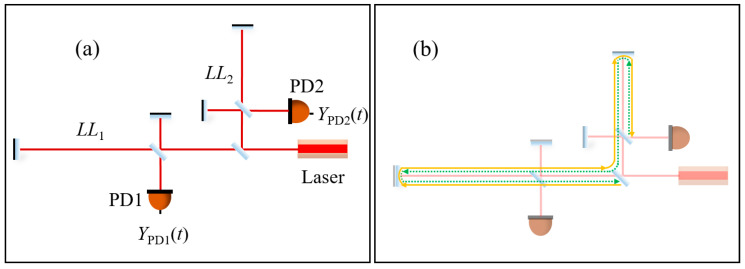
(**a**): Michelson interferometer with different arm lengths. In this case, the laser phase noise cannot be removed directly. PD: photodetector; (**b**): physical meaning of time delay interferometry. The orange solid line and the green dotted line experience the same time delay, and share the same phase noise.

GWs are often very weak at 10^−20^ in the science band. Therefore, the overwhelming noises, such as the laser frequency noise and the clock noise, should be reduced enough to meet the requirement of GW detection. TDI will be used in the post-processing to reduce the noises. Here, we would like to briefly describe the concept of TDI. Figure 2a shows the schematic, and there are two Michelson interferometers with different arm lengths, *LL*_1_ and *LL*_2_, respectively. Two photodetectors, PD1 and PD2, are used to detect the signals.

The signals detected by PD1 and PD2 can be written as:

(1) YPD1(t)=p(t−2LL1c)−p(t)+h1(t)

(2) YPD2(t)=p(t−2LL2c)−p(t)+h2(t)

where *p*(*t*) is the phase noise of the laser source, and *c* is the light speed in vacuum. *h*_1_(*t*) and *h*_2_(*t*) are the GW signals, respectively. The difference between Equations (1) and (2) can be expressed as:

(3) YPD1(t)−YPD2(t)=p(t−2LL1c)−p(t−2LL2c)+h1(t)−h2(t)

Since *LL*_1_ ≠ *LL*_2_, the laser phase noise cannot be canceled. We can shift *Y*_PD1_(*t*) and *Y*_PD2_(*t*) by 2*LL*_2_/*c* and 2*LL*_1_/*c*, respectively, and deduce the subtraction:

(4) YPD1(t−2LL2c)−YPD2(t−2LL1c)=p(t−2LL1c)−p(t−2LL2c)+h1(t−2LL2c)−h2(t−2LL1c)

Finally, we can have the combination *X* as:

(5) X(t)=[YPD1(t)−YPD2(t)]−[YPD1(t−2LL2c)−YPD2(t−2LL1c)]  =h1(t)−h2(t)−[h1(t−2LL2c)−h2(t−2LL1c)]

From Equation (5), the laser noise has been removed, and the GW signals are retained. Figure 2b shows the physical images of the combination *X*, where the yellow solid line and the green dotted line experience the same time delay. To date, scientists have developed various TDI combinations, from version 1.0 to 2.5 [32,33,34] and TDI ranging can provide the distance information between the satellites.

**Figure 3:**
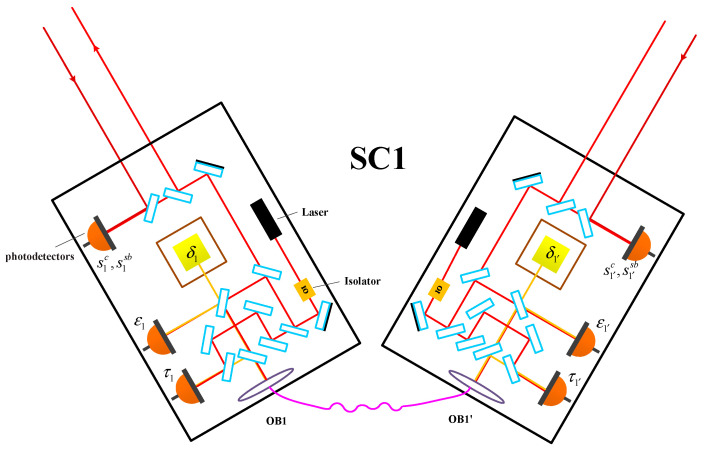
Optical configurations in the spacecraft. Each spacecraft takes two identical optical benches, linked by a piece of fiber. The test mass serves as the inertial reference, and its noise is one part of the noise floor of the whole instrument.

In practice, we do not have such an ideal Michelson interferometer in the constellation. Figure 3 indicates the optical setup in detail, and all the others are identical. Four measurements can be simultaneously obtained for one optical bench, which are the carrier data stream *s^c^*, the side-band data stream *s^sb^*, the test-mass data stream *ε*, and the reference data stream *τ*, respectively. Please note that, in real situations, the side-band data stream is also needed, which can be used to reduce the clock noise. In this work, we focus on the technique of TDI ranging, and the side-band data stream can be disregarded. The data sets can be written as:

(6) $$\begin{array}{l} s_{i}^{c}(t)=h_{i}(t)+D_{i-1}p_{(i+1{)}^{\prime }}(t)-p_{i}(t)+\left[D_{i-1}\Delta_{(i+1{)}^{\prime }}(t)-\Delta_{i}(t)\right]+N_{i}^{\mathrm{opt}}(t)\\ \varepsilon_{i}(t)=p_{i^{\prime }}(t)-p_{i}(t)+\mu_{i^{\prime }}(t)-2\left[\delta_{i}(t)-\Delta_{i}(t)\right]\\ \tau_{i}(t)=p_{i^{\prime }}(t)-p_{i}(t)+\mu_{i^{\prime }}(t) \end{array}$$

and

(7) $$\begin{array}{l} s_{i^{\prime }}^{c}(t)=h_{i^{\prime }}(t)+D_{(i+1{)}^{\prime }}p_{i-1}(t)-p_{i^{\prime }}(t)+\left[D_{(i+1{)}^{\prime }}\Delta_{i-1}(t)-\Delta_{i^{\prime }}(t)\right]+N_{i^{\prime }}^{\mathrm{opt}}(t)\\ \varepsilon_{i^{\prime }}(t)=p_{i}(t)-p_{i^{\prime }}(t)+\mu_{i}(t)-2\left[\delta_{i^{\prime }}(t)-\Delta_{i^{\prime }}(t)\right]\\ \tau_{i^{\prime }}(t)=p_{i}(t)-p_{i^{\prime }}(t)+\mu_{i}(t) \end{array}$$

where *h_i_* is the GW signal, *p_i_* is the laser frequency noise, Δ*_i_* is the optical bench noise, *δ_i_* is the test mass noise, $N_{i}^{\mathrm{opt}}(t)$ is the optical path noise, and *μ_i_* is the fiber noise. The time delay operators *D_i_* are defined as *D_i_p*(*t*) *= p*(*t* − *L_i_*/*c*), where *L_i_* is the baseline length, and *c* is the light speed.

To eliminate the optical bench noise and the primed laser frequency noise, the variables *ξ_i_* and *z_i_* can be introduced as:

(8) $$\begin{array}{l} \xi_{i}(t)\equiv s_{i}^{c}(t)-\frac{\varepsilon_{i}(t)-\tau_{i}(t)}{2}-\frac{D_{i-1}\varepsilon_{(i+1{)}^{\prime }}(t)-D_{i-1}\tau_{(i+1{)}^{\prime }}(t)}{2}\\ \xi_{i^{\prime }}(t)\equiv s_{i^{\prime }}^{c}(t)-\frac{\varepsilon_{i^{\prime }}(t)-\tau_{i^{\prime }}(t)}{2}-\frac{D_{(i+1{)}^{\prime }}\varepsilon_{i-1}(t)-D_{(i+1{)}^{\prime }}\tau_{i-1}(t)}{2} \end{array}$$

and

(9) $$z_{i}=\frac{\tau_{i}-\tau_{i^{\prime }}}{2}$$

Next, the data streams *η_i_* can be written as:

(10) $$\begin{array}{l} \eta_{i}(t)\equiv \xi_{i}(t)-D_{i-1}z_{i+1}\\ \eta_{i^{\prime }}(t)\equiv \xi_{i^{\prime }}(t)+z_{i} \end{array}$$

which are

(11) $$\begin{array}{c} \eta_{i}(t)=h_{i}(t)+D_{i-1}p_{i+1}(t)-p_{i}(t)+\left[D_{i-1}\delta_{(i+1{)}^{\prime }}(t)-\delta_{i}(t)\right]+N_{i}^{\mathrm{opt}}(t),\\ \eta_{i^{\prime }}(t)=h_{i^{\prime }}(t)+D_{(i+1{)}^{\prime }}p_{i-1}(t)-p_{i}(t)+\left[D_{(i+1{)}^{\prime }}\delta_{i-1}(t)-\delta_{i^{\prime }}(t)\right]+N_{i^{\prime }}^{\mathrm{opt}}(t). \end{array}$$

Here, we have got a set of data streams, only involving the GW signals, the laser frequency noise, the test mass noise, and the optical path noise. The secondary noises include the test mass noise and the optical path noise.

Finally, the TDI combinations can be generally written as:

(12) $$\mathrm{TDI}=\sum_{i=1,2,3}\left(P_{i}\eta_{i}+P_{i^{\prime }}\eta_{i^{\prime }}\right)$$

where *P_i_* is the coefficient related to the time delay operators. For the 1st generation *X* combination, we have

(13) $$X_{1}=\left(-1+D_{2^{\prime }2}\right)\eta_{1}+\left(D_{2^{\prime }}-D_{33^{\prime }2^{\prime }}\right)\eta_{3}+\left(1-D_{33^{\prime }}\right)\eta_{1^{\prime }}+\left(D_{2^{\prime }23}-D_{3}\right)\eta_{2^{\prime }}$$

where

(14) $$\begin{array}{l} P_{1}=-1+D_{2^{\prime }2}\\ P_{2}=0\\ P_{3}=D_{2^{\prime }}-D_{33^{\prime }2^{\prime }}\\ P_{1^{\prime }}=1-D_{33^{\prime }}\\ P_{2^{\prime }}=D_{2^{\prime }23}-D_{3}\\ P_{3^{\prime }}=0 \end{array}$$

To date, scientists have developed various TDI combinations, from version 1.0 to 2.0, and several nice reviews can be found [18]. The residual noise after TDI (i.e., the noise floor of the whole instrument) is related to the secondary noise. TDI ranging can provide the distance information between the satellites.

## 3. Principle of Inter-Satellite Ranging for Space-Borne Gravitational Wave Detection

### 3.1. Arm-Locking Ranging

In this section, we describe the principle of TDI ranging, and the schematic is shown in Figure 2a. In the measurement principle, we first use arm-locking ranging to coarsely determine the inter-satellite distance. Arm locking is a technique for laser frequency noise reduction, in which the laser frequency of the cavity-stabilized laser is locked to the baseline length of the constellation [29,30,31]. Since the baseline of the space-borne GW detector is ultra-stable in the science band, the laser frequency noise can be further reduced. To clearly explain the principle, let us go back to Equation (1). We take Laplace Transform (LT) of Equation (1) and neglect the term of GW signals, which gives

(15) $$LT\left(s\right)=P\left(s\right)-P\left(s\right)e^{-s\frac{2LL_{1}}{c}}$$

where *s* is the Laplace variable, and *P* is the transform of *p*(*t*). Therefore, the transfer function *T* can be expressed as

(16) $$T\left(s\right)=1-e^{-s\frac{2LL_{1}}{c}}$$

To evaluate the frequency response, *s* is set to *iω* (i.e., *i*2*πf*). Consequently, Equation (16) is updated to

(17) $$T\left(\omega \right)=1-e^{-i\omega \frac{2LL_{1}}{c}}=2i\sin\left(\omega \frac{LL_{1}}{c}\right)e^{-i\omega \frac{LL_{1}}{c}}=2i\sin\left(2\pi f\frac{LL_{1}}{c}\right)e^{-i2\pi f\frac{LL_{1}}{c}}$$

Considering the amplitude-frequency response of Equation (17), a series of zeros can be found every *c*/(2*LL*_1_) Hz. This means that we can determine *LL*_1_ by the zero position in the amplitude-frequency response, which is the principle of arm-locking ranging. The measurement precision is related to the sample number of the Fourier transform. When increasing the sample number, the measurement resolution can be improved. Considering the requirement of TDI, precision of about 1 m (i.e., 0.3 ns) is needed. Many samples are needed to achieve this level of precision. Despite the fact that the precision of arm-locking ranging could not directly meet the requirement of TDI, it has the advantages of a large measurable range (infinitely large in principle), ease of use, and lack of need for additional hardware. In this case, the system itself can measure the distances between the satellites coarsely, without the need for the deep space network or the orbit data. Please note that the arm-locking loop does not actually need to be closed in this step.

### 3.2. TDI Ranging

Next, we use TDI ranging to finely determine the distances. Take the combination *X* in Equation (5) as an example, and we again neglect the effect of the GW signals. When *LL*_1_ and *LL*_2_ are the exact distance values that the laser noises travel through, Equation (5) should be strictly equal to zero. However, *LL*_1_ and *LL*_2_ cannot be determined so perfectly. In TDI ranging, we can scan the values of *LL*_1_ and *LL*_2_, and find the minimum power of Equation (5). Here, the minimum power of Equation (5) means the integration of the power spectral density in the science band. After finding the minimum power, the instant values of *LL*_1_ and *LL*_2_ are the measured distances between the satellites. The power of Equation (5) can be given by

(18) $$I({\hat{L}}_{k})=\frac{1}{T}\int_{0}^{T}{\left(X({\hat{L}}_{k})\right)}^{2}dt$$

where ${\hat{L}}_{k}$ (*k* = 1,2) is the estimated value of delay time, *X* is the term related to laser noise in Equation (5). When ${\hat{L}}_{k}$ is the right distance value, the above equation can obtain the minimum value. The accuracy of time delay depends on the remaining level of laser noise and the level of secondary noise, as well as the integration time *T*. Basically, TDI ranging is based on the technique of time-of-flight, which means that we can measure the distances by using the flight time of the laser frequency noise. In fact, TDI ranging is a kind of technique for data processing, which aims to find the minimum power of the residual noise after the TDI combinations, by shifting the data streams. When the two data streams are precisely aligned in the time line, the power of the residual noise becomes low. Consequently, the distance information can be extracted from the two data streams.

Due to the large data volume, the scientific data will be down sampled with about 3~10 Hz sampling rate before transmitted to the ground, leading to an error of about 0.33~0.1 s when shifting the data. Therefore, the technique of Fractional Delay Interpolation (FDI) will be used to interpolate the data streams, and then perform the fractional shifts. By convolving the discrete time series with continuous sine basis functions, FDI almost perfectly reconstructs the band-limited signals with extremely low additional noises. More detailed information can be seen in Ref. [35].

## 4. Numerical Simulation of TDI Ranging

In this section, we perform the numerical simulations of TDI ranging. In the simulations, the laser frequency noise is measured by beating two cavity-stabilized lasers in our lab, with 10 Hz sampling rate. The data format is set to double precision floating-point. The data length is 10,000 s, so that the low frequency can reach 0.1 mHz. The simulation schematic is shown in Figure 2a. The laser output is split into two parts, and each part is guided into a Michelson interferometer. We can get two data streams, *Y*_PD1_ and *Y*_PD2_. Please note that we use the combination *X* to verify the performance, and there are plenty of combinations that can be used to perform the TDI ranging.

**Figure 4:**
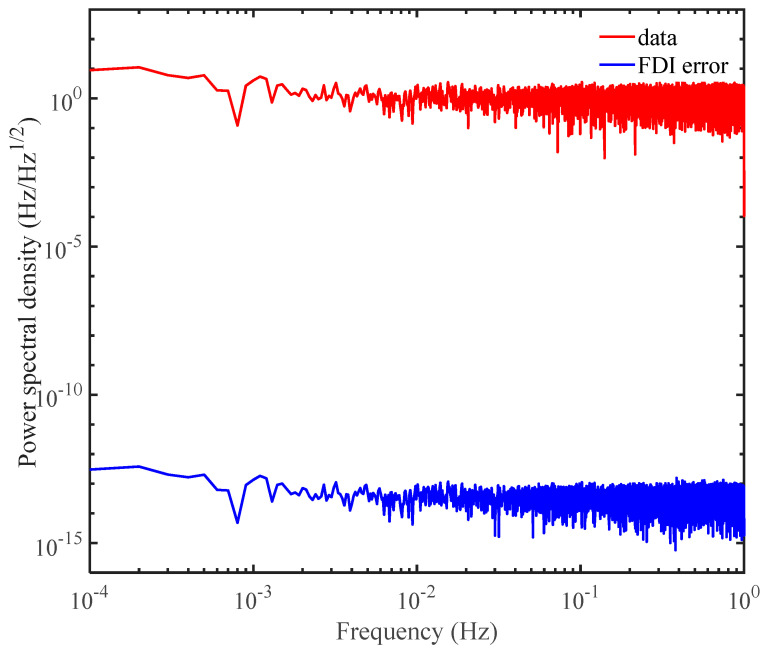
Performance of FDI in the science band. The red line indicates the noise of the raw data at about 10 Hz/Hz^1/2^ in the science band. We use FDI to get the fractional-delayed version of the raw data, and derive the difference between the two versions. The blue line represents the difference, which is well below 10^−12^ Hz/Hz^1/2^.

In practice, the effective sampling rate in the satellite is about 3–5 Hz with the consideration of the communication resource, which can lead to 0.33–0.2 s time error correspondingly. Consequently, the data interpolation is required. In our simulations, the fractional delay interpolation FDI is used. We would like to first examine the performance of FDI, and the results are shown in Figure 4. We use the laser frequency noise simulated by ourselves here, which is about 10 Hz/Hz^1/2^ at 1 mHz, and the sampling rate is 1 kHz. The raw data are first delayed by 0.0015 s and down-sampled with a 10 Hz sampling rate. Then, we use FDI to directly obtain the delayed data (the delay time is 0.0015 s), and compare the two versions. The difference between the two versions is shown in Figure 4, which can be less than 10^−12^ Hz/Hz^1/2^ in the science band. Therefore, the noises caused by the program of FDI are sufficiently small and can be neglected in our simulations.

### 4.1. Arm-Locking Ranging

**Figure 5:**
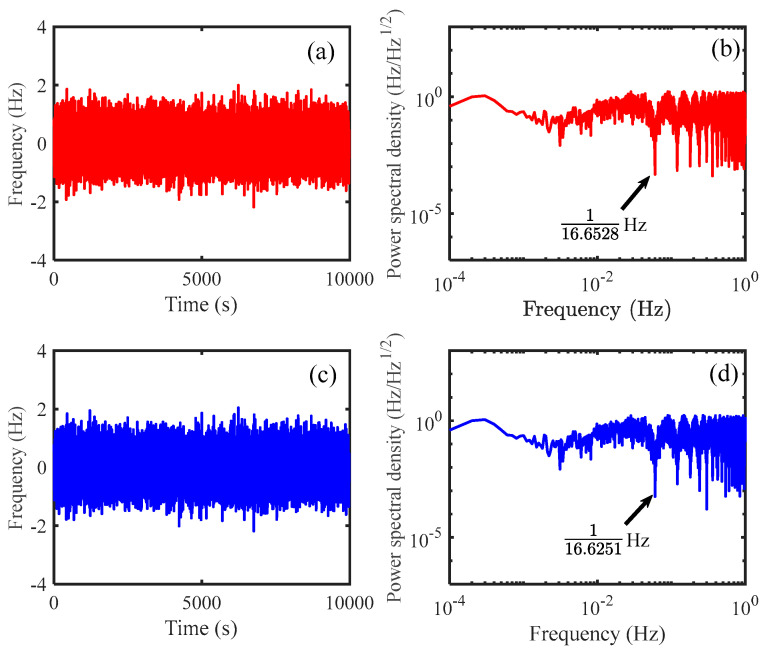
(**a**): The signal of *Y*_PD1_, which is generated by using the laser frequency noise measured in our lab. (**b**): Power spectral density of *Y*_PD1_; a series of zeros can be found along the horizontal axis, which corresponds to the round-trip time delay between the satellite. The delay can be roughly determined in this step. (**c**): The signal of *Y*_PD2_, which is generated by using the laser frequency noise in our lab. (**d**): the power spectral density of *Y*_PD2_. Similarly, we observe a series of zeros, which can be used to measure the distances.

We first use arm-locking ranging to coarsely determine the distances. The data streams *Y*_PD1_ is shown in Figure 5a, where the delay (corresponding to *LL*_1_) we set is 16.674411111111 s, and the noise fluctuation can be within ±2 Hz. It is worth noting that here, 12 decimal places are recommended for the convenience of the performance verification. We transform this data stream, and the power spectral density is indicated in Figure 5b. We find the zeros located about every 0.06 Hz, and the delay can be determined roughly as 16.6528 s. Similarly, we can determine the delay corresponding to *LL*_2_ as about 16.6251 s, as shown in Figure 5c,d, while the delay we set in our simulation is 16.610022222222 s. We find that the distances can be easily determined by simply Fourier transforming the data streams. Please note that the measurement precision is relatively high in the numerical simulations, which could be degraded in real situations.

### 4.2. TDI Ranging

**Figure 6:**
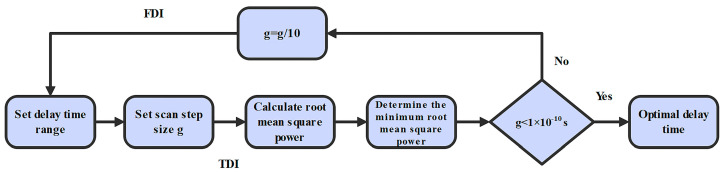
Flow chart of TDI ranging in our simulations. First, we set the scanning range and the step size. Then, we calculate the power of the noise, and find the minimum power. Here, the measurement accuracy can be evaluated. When the accuracy is better than 10^−10^ s, the current delays are the measurement results.

**Figure 7:**
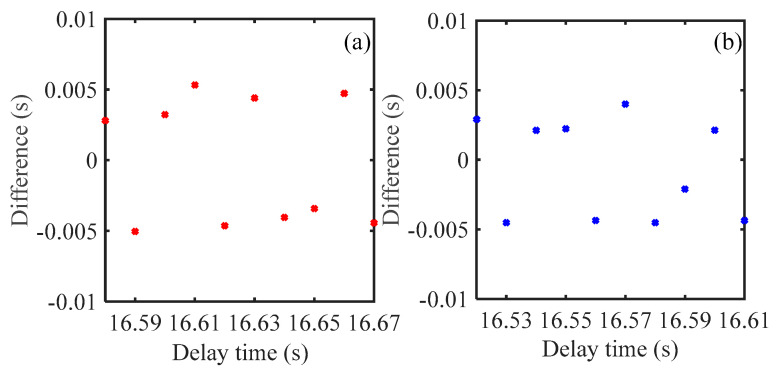
(**a**): Measurement results of *D*_1_. The measurement accuracy can be within ±0.005 s. (**b**): Measurement results of *D*_2_. The measurement accuracy can be within ±0.005 s. *D*_1_ is equal to *LL*_1_/*c*, and *D*_2_ is *LL*_2_/*c*. *c* is the light speed in the vacuum.

**Figure 8:**
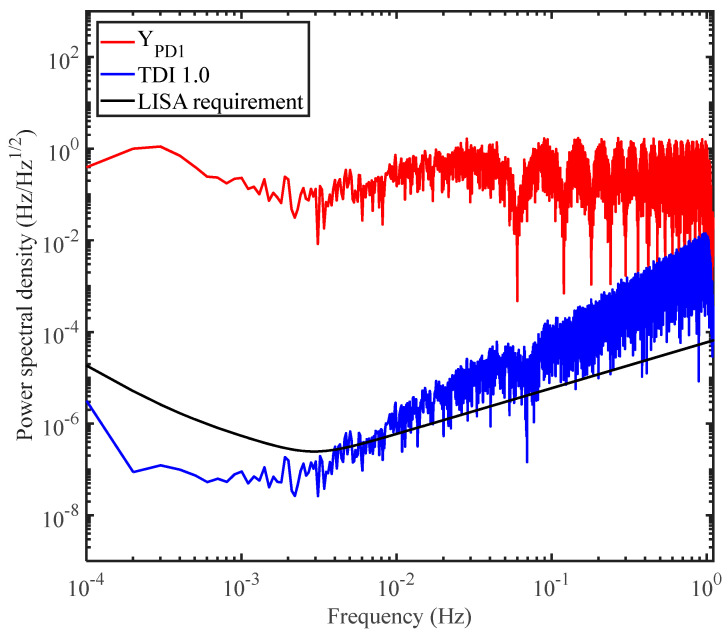
Noises before and after time delay interferometry in our simulations. The red line represents the noise before TDI. The black line indicates the noise floor of the space-borne GW detector, determined by the test mass noise and the shot noise. The blue line shows the noise after TDI. We found, by using the measurement results we obtained, the residual noise could not meet the requirement. This is because the measurement accuracy is not high enough.

Next, we use TDI ranging to finely measure the distances. In TDI ranging, both the delays will be scanned to find the minimum power. After arm-locking ranging, we can give a distance estimation, which can result in a two-dimensional scanning range of (16.6 ± 0.1 s, 16.6 ± 0.1 s). Please note that a broader range is recommended to circumvent the possible misjudgments. Figure 6 shows the flow chart we use in our simulations. The first step is to set the delay parameters as (16.6 ± 0.1 s, 16.6 ± 0.1 s) based on the results provided by the arm-locking ranging. To maintain computing efficiency, we use the strategy of the stepwise scanning. We first set the scanning step size *g* as 0.01 s, and calculate the root mean square power corresponding to all the possible delays. Consequently, we can obtain one set of parameters as (16.67 s, 16.61 s), where the minimum mean square power can be reached. In the meantime, the delays can be determined as 16.67 s and 16.61 s, and the measurement accuracy can be evaluated. If the accuracy is better than 10^−10^ s, the current parameters are the exact delays we need. Otherwise, the scanning range is gradually reset as (16.67 ± 0.01 s, 16.61 ± 0.01 s) with 0.001 s step size by using FDI. Then, repeat the above operation until the delay values with an accuracy better than 10^−10^ s is obtained. Figure 7 shows the results of distance measurements. *D*_1_ is the round-trip delay corresponding to *LL*_1_, and *D*_2_ corresponds to *LL*_2_. We find that the measurement accuracy can be about 0.005 s, which cannot meet the requirement of 0.1 m (about 0.3 ns) in our simulations. We further use the residual noise after TDI to examine the measurement results, as shown in Figure 8. The residual noise cannot meet the requirement of the space-borne GW detection either. The reason we consider this is the laser frequency noise is nearly white noise, which means that the signal to noise ratio is not sufficiently high. When scanning and subtracting the two data streams, the difference is also relatively stable, leading to the position of the minimum power not being so obvious. Consequently, misjudgments could occur, and the distances may not be determined correctly. We suggest using the tone-assisted TDI ranging, which we expect can meet the requirement.

### 4.3. Tone-Assisted TDI Ranging

In the last subsection, we use TDI ranging to determine the distances; however, the performance does not meet the requirement. In this section, we use tone-assisted TDI ranging to improve the measurement performance. Here, we involve a tone signal (i.e., a stable modulation of the laser frequency) in the laser frequency noise, which will be time-shifted to find the minimum power of the residual noise. In our simulations, the modulation depth is 1 kHz, and the frequency is 1 Hz. Compared with the classical TDI ranging, the tone signal takes higher power, implying that the signal to noise ratio can be greatly improved [28]. In particular, we can only focus on the tone signal to carry out TDI ranging, and the noises with other frequencies does not affect the ranging performance. It is worth noting that the tone frequency should not be close to the integer multiple of the round-trip delay time, to avoid peak aliasing. The flow chart of tone-assisted TDI ranging is nearly the same as that in Figure 6. The only difference is that a tone signal is involved in the laser frequency noise. Therefore, the step of filtering out the tone signal is placed in the front end of the program.

**Figure 9:**
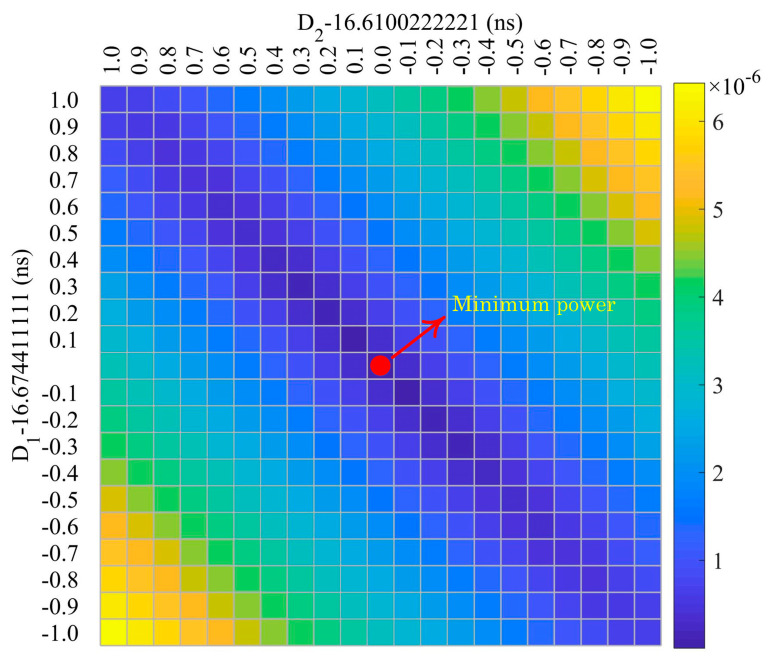
Power distribution after scanning the delays of *D*_1_ and *D*_2_. When scanning the parameters of *D*_1_ and *D*_2_, we obtain a 2D power distribution, which can be used to find the minimum power.

**Figure 10:**
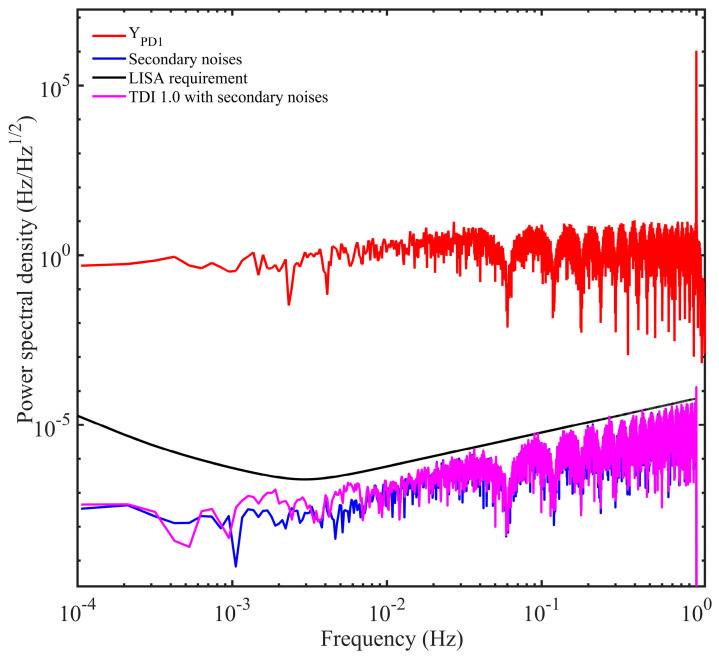
Noises before and after time delay interferometry based on the tone-assisted TDI ranging in our simulations. The red line represents the noise before TDI. The tone signal with 1 Hz modulation frequency can be easily observed. The black line indicates the noise floor of the space-borne GW detector, determined by the test mass noise and the shot noise. The blue line shows the secondary noises. The pink link indicates the noise after TDI. The results based on the tone-assisted TDI ranging have been greatly improved compared with that in Figure 8, meeting the requirement of GW detection.

The power distribution after scanning is shown in Figure 9, and the minimum power appears at the location of (16.674411111 s, 16.6100222221 s). Consequently, we use these values to perform TDI, which are shown in Equation (5), to examine the residual noises. The results are shown in Figure 10, and the residual noises can be around 10^−8^ Hz/Hz^1/2^ in the science band, well meeting the requirement of the space-borne GW detections.

**Figure 11:**
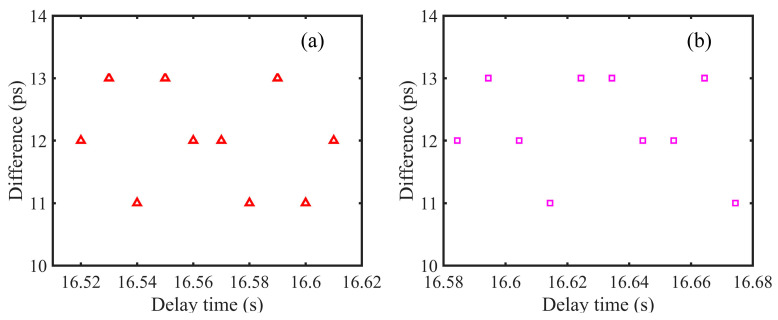
(**a**): Measurement results of *D*_1_. The measurement accuracy is about 12 ps. (**b**): Measurement results of *D*_2_. The measurement accuracy is about 12 ps.

In our simulations, we change the set of *D*_1_ and *D*_2_ with 0.005 s step size and utilize tone-assisted TDI ranging to measure the delays. The measurement results are shown in Figure 11, and the measurement accuracy is about 12 ps. These performances are much better than 0.3 ns (i.e., about 0.1 m), meeting the requirement of gravitational wave detection. We consider that the measurement accuracy in the simulations is limited by the resolution of the software, not the method itself. Please note that, we only discuss the 1st generation *X* channel in this work. We consider that, the 1st generation Michelson combination is more practical in inter-satellite ranging. The *X* combination can be used to measure two arm lengths in the constellation, and the *Y* combination can be utilized to determine another arm length. In this case, the three arm lengths can be determined, which can be used by the other TDI combinations.

In the case of the 2nd generation TDI ranging, we actually tested the performance in the numerical simulations. Here, the data are divided into several segments, and each segment can be used to derive a measured distance. Then, the distance variation can be obtained. In our simulations, we find that the data length of 100 s is sufficient to precisely determine the distances.

## 5. Experimental Demonstration of Tone-Assisted TDI Ranging

**Figure 12:**
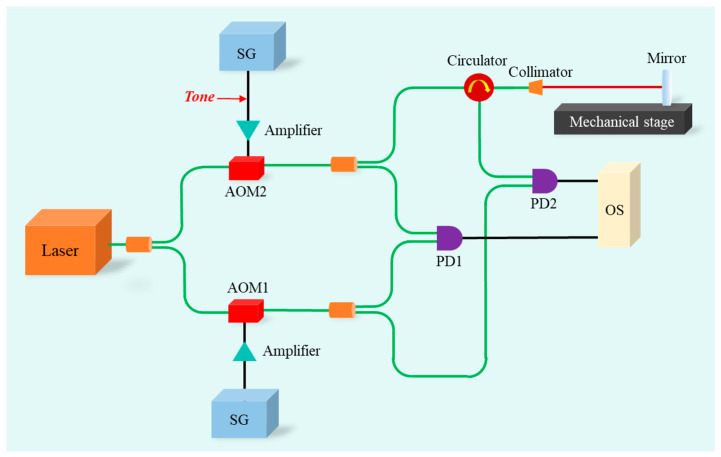
Experimental setup of the tone-assisted TDI ranging. AOM: acousto-optic modulator; PD: photodetector; SG: signal generator; OS: oscilloscope.

In this section, we carry out the experiments of the tone-assisted TDI ranging, and the experimental setup is shown in Figure 12. The output of the laser source (Koheras AdjustiK Y10, 1542 nm wavelength, 30 mW average power) is split into two parts. One part after an acousto-optic modulator AOM2 (AAopticsG-1550-150) serves the local oscillator. The other part working as the signal source is frequency shifted by an acousto-optic modulator AOM1 (AAoptics G-1550-150), and the tone signal (30 kHz frequency, and 17 MHz modulation depth) is encoded into the driving frequency of AOM1. Then, the signal source is again split into two parts: the reference arm and the measurement arm. The reference arm is directly combined with the local oscillator. In the measurement arm, the laser after the optical circulator is guided to a mirror fixed on a movable stage and finally combined with the local oscillator. Two photodetectors are used to detect the heterodyne signals corresponding to the reference and measurement arm, respectively. Both signals are measured and stored by an oscilloscope (LeCroy HDO6104A). The technique of tone-assisted TDI ranging is used to determine the distances.

**Figure 13:**
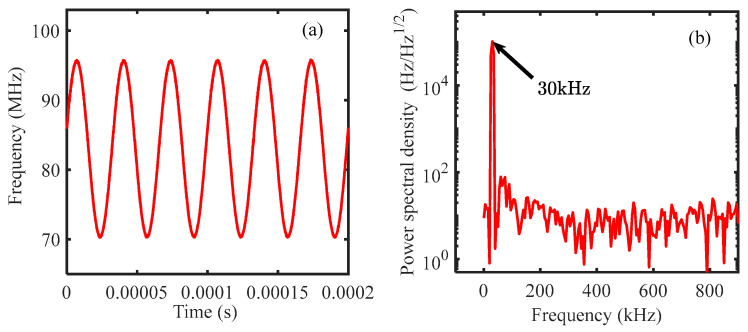
(**a**): Tone signal in our experiments. The modulation depth is 17 MHz, and the modulation frequency is 30 kHz; (**b**): Power spectral density of the tone signal. A peak at 30 kHz is clearly observed.

Figure 13 shows the tone signal we use in our experiments, which is a stable sine signal. We use TDI ranging to determine the distances. Please note that one-dimensional scanning is sufficient in this experiment. When scanning the time delay, the power of the signal keeps changing, and a minimum value exists, which corresponds to the distance difference between the reference and measurement arms.

**Figure 14:**
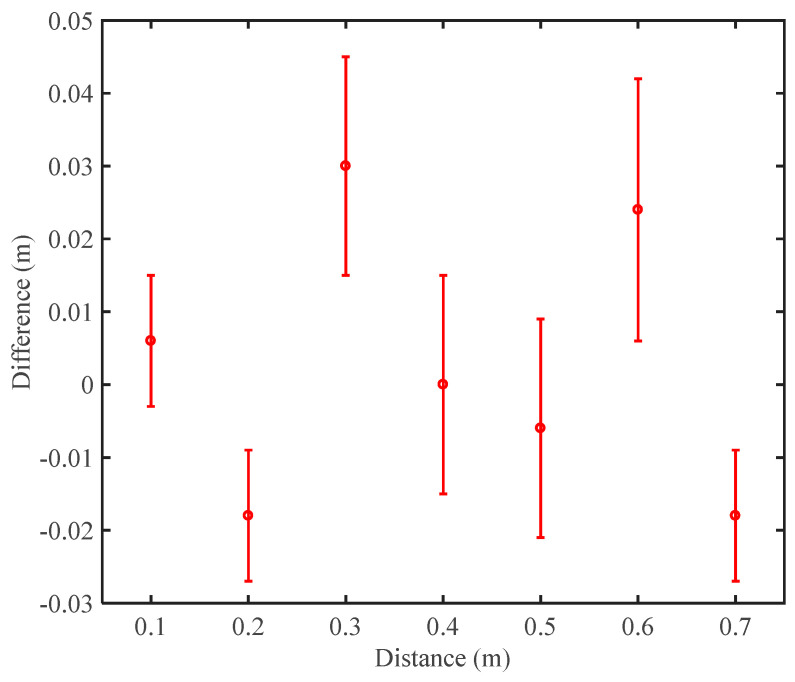
Results of the distance measurement using tone-assisted TDI ranging. In our experiments, the measurement uncertainty can be within ±0.05 m. The red circles show the average values of five single measurements, and the error bar indicates the standard deviation.

In the experiments, the measurement mirror is fixed on a movable stage. The stage is moved by a step size of 0.1 m, and at each position the distances are measured five times. The precision of the movable stage is about 50 μm, which can be used as the reference to evaluate the performance of TDI ranging. The results of the distance measurement are indicated in Figure 14. The comparison with the reference values shows a difference within 0.05 m, better than 0.1 m, which can meet the requirement of TDI. We consider that the laser frequency noise, the electrical noises, and the environment instability can make contributions to the final results.

## 6. Conclusions

In this work, we numerically and experimentally demonstrate that TDI ranging can realize high-precision distance measurement between satellites, and no additional hardware is needed. In particular, we describe a new strategy for inter-satellite ranging in future space-borne GW detectors. The distances can be coarsely determined by arm-locking ranging with a large non-ambiguity range, and then TDI ranging can be utilized to finely measure the distances with better than 0.1 m precision. Our simulation results show the precision can reach a millimeter level when the delay is about 16.6 s by using tone-assisted TDI ranging. To verify the performance in the real optics, we built a simple system, and the experimental results show the measurement uncertainty can be well below 0.05 m, meeting the requirement of TDI.

## Data Availability

The data can be obtained upon reasonable request to the corresponding author.
